# Influencing factors of medication adherence in schizophrenic patients: a meta-analysis

**DOI:** 10.1038/s41537-023-00356-x

**Published:** 2023-05-15

**Authors:** Jing Guo, Xue Lv, Yan Liu, Lingling Kong, Haiying Qu, Weihua Yue

**Affiliations:** 1 Department of Psychology, Medical Humanities Research Center, Binzhou Medical University, Yantai, 264003 China; 2 Peking University Sixth Hospital, Peking University Institute of Mental Health, Beijing, 100191 China; 3 National Clinical Research Center for Mental Disorders (Peking University Sixth Hospital), Beijing, 100191 China; 4 NHC Key Laboratory of Mental Health (Peking University), Beijing, 100191 China; 5 The First Affiliated Hospital of Xinxiang Medical College, Xinxiang, Henan 453100 China; 6 PKU-IDG/McGovern Institute for Brain Research, Peking University, Beijing, 100871 China; 7 Chinese Institute for Brain Research, Beijing, 102206 China

**Keywords:** Schizophrenia, Psychiatric disorders

## Abstract

Medication adherence of schizophrenic patients is a growing public health problem. We conducted a meta-analysis on the influencing factors of medication compliance in schizophrenic patients. We searched PubMed, Embase, Cochrane Library, and Web Of Science for relevant articles published up to December 22, 2022. Combined odds ratios (ORs) and 95% confidence intervals (CIs) were used to assess influencing factors. Egger’s test, funnel plot, the trim and fill method, and meta-regression analysis were used to assess publication bias. A total of 20 articles were included in the analysis. Twenty influencing factors were divided into seven categories: drug factors (OR = 1.96, 95% CI: 1.48–2.59), problem behavior (OR = 1.77, 95% CI: 1.43–2.19), income and quality of life (OR = 1.23, 95% CI: 1.08–1.39), personal characteristics (OR = 1.21, 95% CI: 1.14–1.30), disease factors (OR = 1.14, 95% CI: 1.98–1.21), support level (OR = 0.54, 95% CI: 0.42–0.70), and positive attitude and behavior (OR = 0.52, 95% CI: 0.45–0.62). This meta-analysis found that drug factors, disease factors, problem behavior, low income and quality of life, and factors related to personal characteristics appear to be risk factors for medication adherence in people with schizophrenia. And support level, positive attitude and behavior appear to be protective factors.

## Introduction

Schizophrenia is a common severe mental disease with a lifelong prevalence rate of 1%, which is mainly manifested in the disorder of mental and psychological processes such as thinking, perception, self-experience, cognition, will, emotion and behavior, and has the characteristics of high disability rate, repeated illness and prolonged course^1,2^. The pathogenesis of schizophrenia is complex and not clear at present. Genetic susceptibility, abnormal neurotransmitter function and external factors may all contribute to the development of schizophrenia^3^.

The current treatment of schizophrenia is based on the administration of antipsychotic medication. Medication can control the condition of schizophrenic patients, which has been effective in the alleviation of positive symptoms, prevention of relapse, and extension of life expectancy^4–6^. The extent to which patients follow the prescribed time intervals and dosage requirements is defined as medication adherence^3,7,8^. A Low degree is considered as poor medication adherence. Medication non-adherence behaviors include not taking medication on time, not taking more or less medication according to the dose, stopping the medication and reducing medication by themselves.

Good medication adherence is the key to the effectiveness of drug treatment. Poor medication adherence will cause many problems in schizophrenic patients^9,10^. First, low medication compliance will lead to low efficacy and high relapse. Some articles pointed out that the symptoms of schizophrenia developed the fastest in the five years before the onset of schizophrenia, dosing on time and at the right dose is an important factor in the effectiveness of treatment, irregular medication is the key risk factor for relapse^11^. In a retrospective analysis of 419 psychiatric inpatients readmitted to hospital, Barnett et al. found that patients’ medication non-adherence significantly increased the risk of readmission compared to other factors, with an OR of 3.33^12^; data from Verdoux et al. show that the most common cause of relapse and readmission in adult studies is the discontinuation of prescription drugs. Patients who do not insist on medication are six times more likely to be admitted to the hospital again than those who insist on medication^13,14^. Furthermore, many studies have shown that poor medication adherence is associated with problematic behaviors such as violence, self-harm, and suicide. Buchanan et al. found that there was a significant positive correlation between low drug compliance and harmful violence through 18-month follow-ups of 1435 schizophrenic patients^15^; Díaz-Fernández et al. pointed out that the lack of treatment compliance of schizophrenic patients is a risk factor for the increase of suicidal behavior^16^; the results of a multicenter clinical trial in China also show that the aggression risk of schizophrenia patients is related to non-adherence to medication^17^.

Through a review of medication non-adherence behavior in schizophrenia patients, Lacro et al. noted that current medication non-adherence in schizophrenia patients is as high as 40–50%^18^. Fenton et al. reported a median non-adherence rate of 55% for oral antipsychotics^19^ and other data in the literature on medication non-adherence in schizophrenia patients ranging from 20 to 89%^20–22^. In recent years, many studies have been reported on the phenomenon of poor medication adherence in patients with schizophrenia, while the factors influencing medication adherence have also become a subject of scholarly interest. Available clinical observations and cohort studies suggest that patients’ age, gender, marital status, insight, other physical illnesses, economic conditions, etc. all have an impact on medication adherence^18,23–25^.

However, the current research lacks uniform predictors and has no clinical implications for interventions for medication adherence in patients with schizophrenia. Understanding the factors that influence medication adherence has practical implications for the identification of patients with low adherence and may assist in the development of clinical interventions for medication adherence. The aim of this study was to analyze the influencing factors of medication adherence of schizophrenic patients, to provide information for the formulation of intervention strategies for medication adherence, and to help patients recover better and reduce recurrence.

## Methods

### Search strategy and inclusion criteria

We conducted a systematic review and meta-analysis according to the Preferred Reporting Items for Systematic Reviews and Meta-Analyses (PRISMA) guidelines. The initial study protocol was preregistered at PROSPERO (CRD42023388661). We searched PubMed, Embase, Cochrane Library, and Web of Science for relevant literature up to 22 December 2022.

The search consisted of the following terms as Medical Subject Headings (MSH) and different databases have corresponding modifications. The following search strategy was used: (schizo*) AND (adherence OR compliance OR non-adherence OR non-compliance) AND (relative OR risk*) AND (cohort OR “cohort study”).

Two researchers independently assessed the title and abstract that met the potential qualifications of the above search strategy to exclude the following articles: (i) use of long-acting injectable (LAI) antipsychotics; (ii) no relevant diagnosis of schizophrenia; (iii) review articles, comments, case reports, editorials, animal studies, and meta-analysis; (iv) data unextractable or incomplete; (v) language not English. The full text was then further assessed to determine whether the article met the inclusion criteria. The final articles included met the following criteria: (i) patients diagnosed with schizophrenia who are receiving at least one antipsychotic drug, and excluded patients with major medical conditions (such as liver or kidney dysfunction in relationship to cardiovascular disease organic brain disorders), with a history of substance use disorders; (ii) the study was designed as a cohort study or a cross-sectional study; (iii) the hazard ratios (HR), relative risks (RR), and odds ratios (OR) were reported.

### Data extraction and quality assessment

All data were extracted independently by two investigators and the following data were collected for all articles: author, year of publication, country, sample size, sample age, diagnostic criteria, and influencing factors for medication adherence.

In terms of quality assessment, we used the Agency for Healthcare Research and Quality Checklist^26^ to assess the cross-sectional studies, and The Newcastle Ottawa Scale (NOS)^27^ was used to assess the cohort study. For each score, 1 indicates that the study meets this criterion and 0 indicates that the study does not meet this criterion. A total score of no less than 5 will be included in this meta-analysis.

### Statistical analysis

In this meta-analysis, Stata 14.0 was used for data analysis^28^. We used the *Q*-test and *I*-squared (*I*^2^) values to assess heterogeneity. *P* < 0.1 and *I*^2^ > 75% was considered significant heterogeneity. *I*-squared (*I*^2^) values close to 0% and *P* > 0.1 indicate that heterogeneity is small. Sensitivity analysis was carried out for the parts with large heterogeneity. Given the expected heterogeneity, we adopted the random effect model in advance. The odds ratio (OR) was used as the main index, and a 95% confidence interval (95% CI) was used to reflect the uncertainty of point estimation, with ORs = 1 indicating that the factor was not associated with medication adherence, ORs < 1 considered as protective factors and ORs > 1 as risk factors.

For publication bias, we used funnel plots for rough qualitative analysis combined with Egger’s test for quantitative analysis, significant Egger’s test result (*P* < 0.05) indicated the presence of publication bias. For results showing publication bias, we used the trim and fill method to assess the impact of bias on final outcomes. In addition, to consider potential moderating effects, we used meta-regression to assess the country of study, the type of population (first patients or not), the type of study (cohort/cross-sectional study), and the quality of the literature.

## Results

### Search results and character of studies

**Fig. 1:**
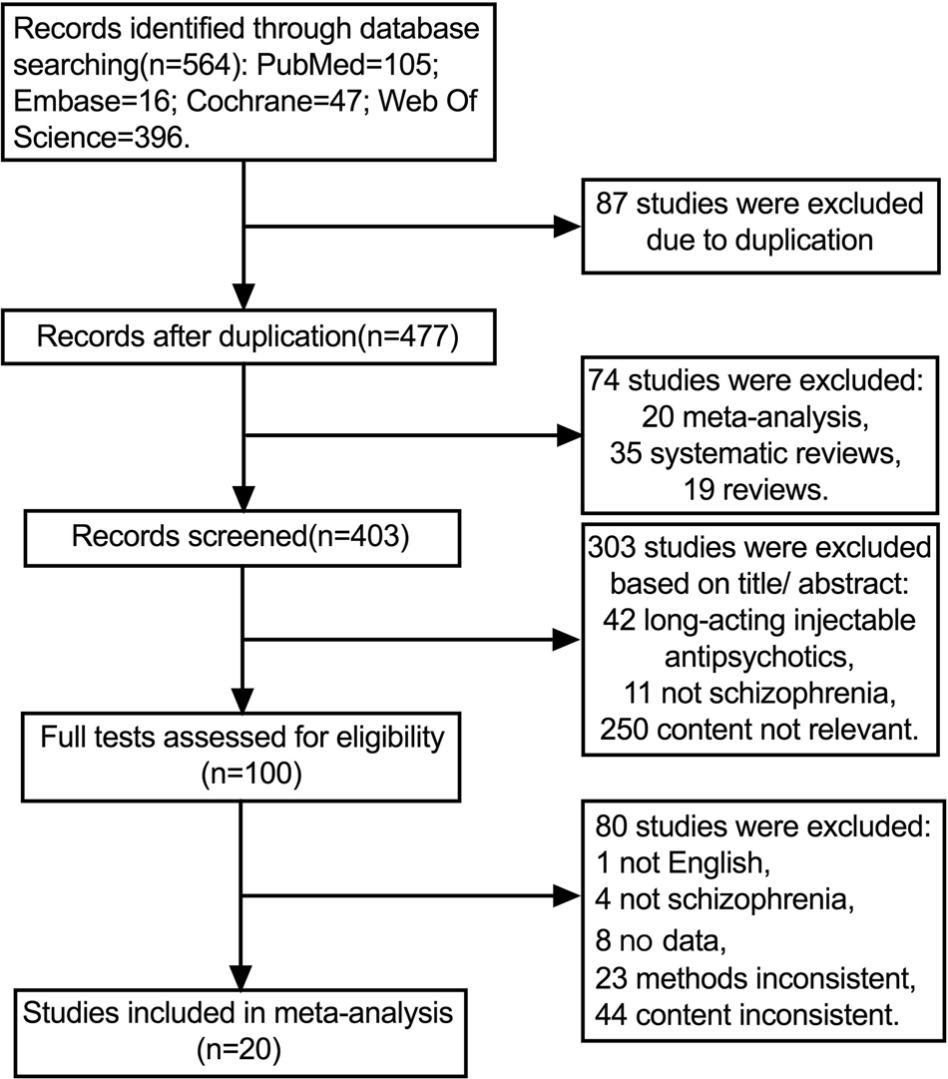
Flow chart of the search for relevant references. The figure represents the amount of literature excluded/included after each step.

A total of 564 articles were retrieved in the initial stage, and 477 articles were left after deleting duplicates. The titles and abstracts were initially screened to remove articles that were not relevant to the content of the review articles. After reading the full text of 100 articles, a total of twenty articles were included in this meta-analysis (Fig. 1).

Of the 20 studies eventually included, five were cross-sectional studies, and the remaining fifteen were cohort studies. The age range was 14–65 years and the diagnostic criteria for schizophrenia were DSM or ICD. The relevant information extracted for the included literature is presented in Table 1. A total of 20 studies had two quality assessment scores of 8, 9 scores of 7, 7 scores of 6, and 2 scores of 5. All met the inclusion criteria, the specific quality assessment scores see Supplementary Tables 1 and 2.

**Table 1:** Characteristics of studies included in the meta-analysis.

Author	Year	Study Design	Sample size *N*	Sample age M (SD)	Period	Diagnostic criteria	Influencing factors	References
Olfson, M.	2000	Cohort study	74	34.8 ± 9.7	18 months	DSM-IV	Substance abuse, past non-compliant behavior, family support	^24^
Acosta, F. J.	2009	Cohort study	102	42.4 ± 11.7	3 months	ICD-10	Insight, disease severity	^47^
Gearing, R. E.	2009	Cohort study	65	15.35 ± 2.08	at least 2 years	DSM-IV	Number of drugs	^48^
Hill, M.	2010	Cohort study	104	29.1 ± 12.0	4 years	DSM-IV	Alcohol and drug abuse, duration of illness	^49^
Lambert, M.	2010	Cohort study	605	15–29	18 months	DSM-IV	Number of medications, severity of illness, insight	^50^
Conti, V.	2012	Cohort study	4975	46.4 ± 15.3	1 years	ICD-10	Employment, city life, marriage, hospitalization	^51^
Hui, C. L. M.	2016	Cohort study	313	38.2 ± 8.4	6 months	DSM-IV	Severity of illness, hospitalization at onset, social support, attitudes to treatment	^52^
Anderson, J. P.	2017	Cohort study	308	/	1 years	DSM-IV	Psychological interventions, age, family support, alcohol and drug abuse, gender, education level, attitudes to treatment, social support	^53^
Conus, P.	2017	Cohort study	584	22.1 ± 3.4	18 months	DSM-IV	Severity of illness, level of education, social support	^54^
Daneault, J. G.	2019	Cohort study	421	14–35	2 years	DSM-IV	Alcohol or drug abuse, severity of illness	^55^
Desai, R.	2019	Cross-sectional study	496	/	/	ICD-9	Gender, family support, level of education	^56^
Rezansoff, S. N.	2019	Cohort study	1996	35.4 ± 9.4	17 months	ICD-9	Attitude to treatment, amount of medication	^57^
Tan, C. Z.	2019	Cohort study	445	26.3 ± 6.6	1 years	DSM-IV	Gender, family support, self-knowledge	^58^
Wang, D.	2020	Cross-sectional study	1189	34.22 ± 12.29	/	DSM-IV	Age, income, hospitalized at onset, severity of illness	^59^
Guitter, M.	2021	Cohort study	136	29.5 ± 9.9	1 years	ICD-10	Single, family support, hospitalized, other physical illness, active treatment	^60^
Rubio, J. M.	2021	Cohort study	512	/	8 years	ICD-10	Female, first hospitalization, other physical illness, age <25, substance abuse	^61^
Stockbridge, E.	2021	Cross-sectional study	1778	18–64	/	ICD-10	Age >50, education, level of mental health, substance abuse, other physical illness	^62^
Verdoux, H.	2021	Cross-sectional study	326	32.9 ± 0.4	/	DSM-V	Number of drugs, efficacy, side effects, quality of life, psychological interventions, level of education, self-knowledge	^63^
Cho, S. J.	2022	Cohort study	326917	/	6 months	ICD-10	Sex, hospitalized at onset, income	^64^
Kirchner, S. K.	2022	Cross-sectional study	1062	42.82 ± 12.98	/	DSM-IV	Attitude to treatment, medication dose, quality of life, alcohol or drug abuse	^65^

Twenty articles reported twenty influencing factors of medication adherence, such as the number of medications taken, medication effectiveness, medication side effects, duration of illness, the severity of disease, insight, education level, gender, age, and social support level. There was high heterogeneity between factors (*I*^2^ = 95.6% > 75%, *Q*-test: *P* = 0.01 < 0.1), and very significant. Therefore, we divided the different influencing factors into seven categories and discussed them respectively (Table 2).

**Table 2:** Risk of all factor analysis of included studies in this meta-analysis.

Influencing factor	No. of studies	Heterogeneity	Pooled OR	95% CI	Pooled effect size test	
		*I*^2^ (%)			*Z*	*P*
Drug factors	8	85.0	1.96	1.48–2.59	2.59	0.001
Disease factors	14	75.4	1.14	1.98–1.21	4.55	0.001
Personal factors	13	86.4	1.21	1.14–1.30	5.75	0.001
Support level	10	62.1	0.54	0.42–0.70	4.60	0.001
Positive attitudes and behavior	14	88.5	0.52	0.45–0.62	7.94	0.001
Problem behaviors	8	65.9	1.77	1.43–2.19	5.26	0.001
Income and quality of life	6	73.5	1.23	1.08–1.39	3.23	0.001
Overall (risk)	73	89.4	1.13	1.25–1.37	11.76	0.001
(Protective)		83.2	0.54	0.47–0.61	9.44	0.001

### Drug factors

**Fig. 2:**
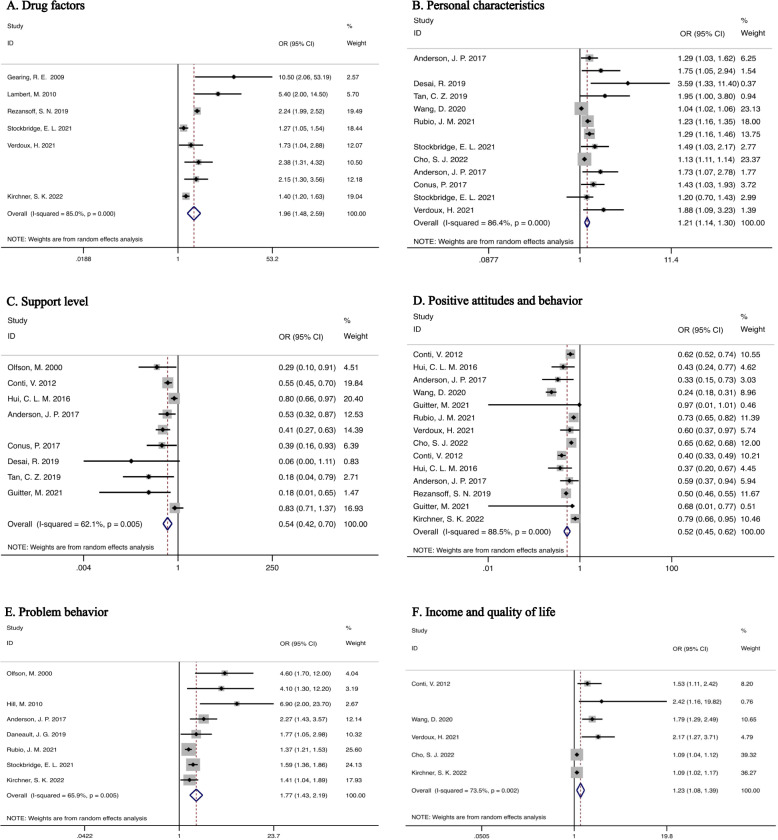
Forest plots for medication factors, personal characteristics, level of support, positive attitudes and behaviors, problem behaviors, income and quality of life. **A** Forest plot of effect sizes and 95% CI for medication factors (random effects model). **B** Forest plot of effect sizes and 95% CI for personal characteristics factors (random effects model). **C** Forest plot of effect sizes and 95% CI for support levels (random effects model. **D** Forest plot of effect sizes and 95% CI for positive attitudes and behaviors (random effects model). **E** Problem behaviors. **F** Effect sizes for income and standard of living and forest plots with 95% CI (random effects model). Diamonds represent individual studies and combined effect sizes, and these lines represent 95% confidence intervals for each main study. OR odds ratio.

Data from a total of eight studies containing the number of drugs taken, the therapeutic effect of drugs, and the side effects of drugs are classified as drug factors. Meta-analysis using randomized effects showed that drug factors were the risk factors of medication adherence in schizophrenic patients (OR: 1.96, 95% CI: 1.48–2.59, *I*^2^ = 85%, *P* < 0.05) (Fig. 2A). Sensitivity analyses showed little effect of removing either literature on the analysis, indicating reliable arithmetic for random effects, indicating that the operation results of random effects are reliable Egger’s test showed that there was no publication bias (*P* = 0.54 > 0.05).

### Disease factors

**Fig. 3:**
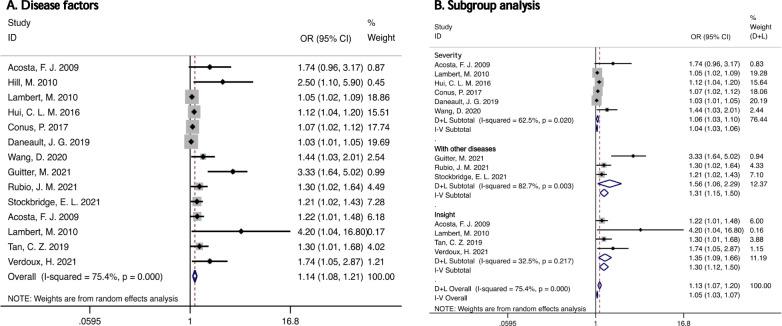
Forest plots and subgroup analysis of disease factors. **A** Forest plot of effect size and 95% CI of disease factors (random effects model). **B** Forest plot for subgroup analysis of disease factors (disease severity, other physical illness, self-knowledge). The diamonds represent individual studies and pooled effect sizes, and the lines represent 95% confidence intervals for each main study. OR odds ratio.

A total of fourteen data items were included in the analysis of disease factors, including elements such as duration of disease, severity of disease, level of insight, and other physical illnesses in combination. The results of the meta-analysis based on random effects showed that the related factors of the disease would cause the non-compliance with medication to a certain extent (OR: 1.41, 95% CI: 1.08–1.21, *I*^2^ = 75. 4, *P* < 0.05) (Fig. 3A). Subgroup analysis of various factors showed that other physical diseases (OR: 1.31, 95% CI: 1.15–1.50) and insight (OR: 1.30, 95% CI: 1.12–1.50) had a great influence on medication compliance (Fig. 3B).

### Personal characteristics

A total of thirteen studies with data on age, gender, education level, and other factors in personal characteristics were included. Meta-analysis showed that there was a significant correlation between individual factors and medication compliance (OR: 1.21, 95% CI: 1.14–1.30, *I*^2^ = 86.4%, *P* < 0.05) (Fig. 2B).

### Support level

The level of support mainly includes social support and family support and involves ten sets of data. Meta-analysis showed that support level was a protective factor for medication adherence in schizophrenic patients (OR: 0.54, 95% CI: 0.42–0.70, *I*^2^ = 62.1%, *P* < 0.05), that is, the higher the level of support, the better the medication adherence (Fig. 2C).

### Positive attitudes and behavior

Fourteen studies were included in the category of positive attitudes and behaviors. Positive treatment attitude refers to the degree of cooperation with physician treatment, and positive behaviors included immediate hospitalization and regular psychological intervention at the onset of the disease. The combined OR value was 0.52 (95% CI: 0.45–0.62, *I*^2^ = 88.5%, *P* < 0.05), which indicated that positive treatment attitude and behavior were protective factors of medication adherence (Fig. 2D).

### Problem behavior

Eight data sets on alcohol and substance abuse are included in the analysis of problem behavior. Results of a meta-analysis based on a random effects model showed that problem behavior was a risk factor for medication adherence (OR: 1.77, 95% CI: 1.43–2.19, *I*^2^ = 65.9%, *P* < 0.05) (Fig. 2E).

### Income and quality of life

A randomized meta-analysis of six groups of data showed that low income and low quality of life were risk factors for medication compliance in schizophrenic patients (OR: 1.23, 95% CI: 1.08–1.39, *I*^2^ = 73.5%, *P* < 0.05) (Fig. 2F).

### Publication bias

**Fig. 4:**
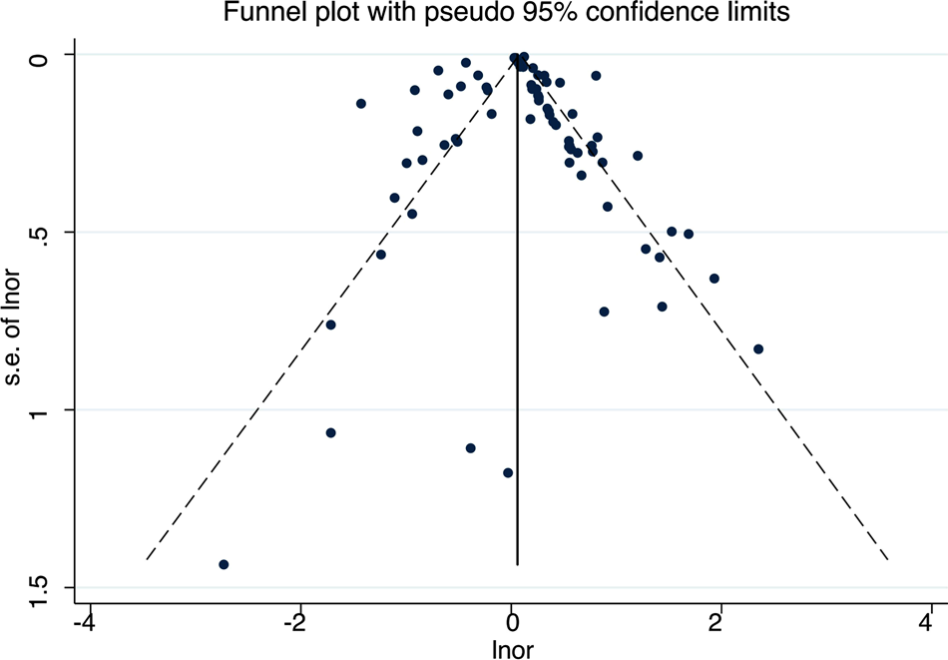
Overall funnel plot. The dotted lines represent 95% confidence intervals, and the dots represent studies.

Funnel plots were conducted for the seven factor categories and the scatter distribution in the funnel plots was asymmetrical for all factors except positive attitudes and behaviors, suggesting that there may be publication bias. Then we further carried out Egger’s test on the factor categories and found that all the other five factors except drug factor (*P* = 0.538) and positive attitude and behavior (*P* = 0.204) had publication bias (*P* < 0.05) (Table 3), which may be due to publication bias. Supplementing the virtual literature with publication bias for the five factors that had publication bias by a trim and fill method revealed very stable results for illness factors, personal traits, level of support and problem behavior, indicating that publication bias had little effect on the study results. In contrast, the results for income and quality of life were not stable, suggesting that publication bias had an impact on the results of the meta-analysis and that more studies need to be included in this factor to reduce publication bias. In addition, we did an overall Egger’s test for all factors and found that the test results were not significant (*P* = 0.976 > 0.05), suggesting that there was no publication bias (Fig. 4).

**Table 3:** Publication bias test.

Influencing factors	*P*
Drug factors	0.538
Disease factors	0.001
Personal factors	0.044
Support level	0.016
Positive attitudes and behaviors	0.204
Problem behaviors	0.003
Income and quality of life	0.012
Overall	0.976

In addition to the funnel plots and Egger’s test, we conducted a meta-regression analysis with the study country, population type (whether first-episode patients), study type and literature quality as variables to further explore the possible sources of heterogeneity. The results showed that population type (*b* = 0.87, *P* = 0.49), research type (*b* = 0.89, *P* = 0.50), and literature quality (*b* = 1.08, *P* = 0.49) were not significant sources of heterogeneity. Study countries (*b* = 3.80, *P* = 0.03) may be a source of heterogeneity. Therefore, we conducted a subgroup analysis of the study countries. A total of 12 countries were involved in this meta-analysis, and the results of the subgroup analysis showed that only two countries had low subgroup heterogeneity (*I*^2^ < 50%, *P* > 0.1), while the majority of countries still had high subgroup heterogeneity (Supplementary Table 3). This suggests that the study country moderates heterogeneity to some extent, but is not itself a significant source of heterogeneity.

## Discussion

It has been noted that the indirect, direct and readmission costs associated with schizophrenia amount to tens of billions of dollars per year^29–31^, and more and more related costs are considered to be related to non-adherence to medication^32^. As early as 1999, the National Center for Mental Health Research emphasized the importance of treatment compliance among patients with mental illness^33^. Although some studies have pointed out that long-acting injectable drugs are an effective way to improve patient adherence to medication^34^, it has certain individual tolerance problems^35^ and the high price will bring great economic pressure on patients^36,37^.

Understanding the factors related to medication compliance can guide us to screen compliance assessment tools and develop practical interventions to better help patients recover to the maximum extent. To our knowledge, this is the first meta-analysis to explore the factors influencing medication adherence in the schizophrenia population.

This study was based on a meta-analysis of five cross-sectional studies and fifteen cohort studies with an overall sample of 342,408 patients taking antipsychotic medication. The data of meta-analysis showed that a total of five categories of factors were potential risk factors for medication adherence, with a combined OR of 1.31 (95% CI: 1.25–1.37, *I*^2^ = 89.4, *P* < 0.05): drug factors (multiple drug doses, poor drug efficacy, high medication side effect response), disease factors (long duration of disease, high severity of disease, combined with other physical diseases, weak insight), personal characteristics (age > 50, age < 25, female, with low education level), problem behavior (alcohol abuse, drug abuse), lower income, and lower quality of life. Two categories of factors were protective for medication adherence, with a combined OR of 0.54 (95% CI: 0.47–0.61, *I*^2^ = 83.2, *P* < 0.05): higher levels of support (family support, social support), and positive treatment attitudes and behaviors.

Among the results of the analysis of risk factors, medication factors had the greatest impact on medication adherence in patients with schizophrenia (OR = 1.96). The results suggest that the therapeutic effect, dosage, and severity of side effects of drugs will lead to patients’ non-compliance behavior. This is consistent with previous research results^38,39^. Therefore, clinicians should combine these dimensions in the selection of patients’ therapeutic drugs to ensure maximum adherence. At the same time, due to the lack of literature, this paper has not analyzed the types of drugs. This is also the limitation of this article. Some studies have shown that adherence to medication is higher with clozapine^40–42^ and risperidone^24^ compared to other antipsychotics, and that adherence is higher with atypical antipsychotics than with typical antipsychotics^21,43,44^. This suggests that the choice of medication type affects patients’ medication adherence, and future analyses could extend this section of the data to explore this further.

In addition to drug factors, the results of our meta-analysis showed that disease-related factors such as problem behaviors of drug and alcohol abuse (OR = 1.77), duration and severity of illness (OR = 1.14) can also influence medication adherence to a large extent. This is consistent with the findings of several previous studies^41,45,46^. Also, lower income and quality of life (OR = 1.23), personal attributes including age, gender, and education (OR = 1.21), were all significant in the results of this meta-analysis and were among the risk factors for patient medication adherence. This is different from the results of Lacro et al.^18^, which may be caused by different inclusion criteria and different data processing methods.

In terms of protective factors for medication adherence, we found two significant results: support level (OR = 0.54), positive attitude and behavior (OR = 0.52). Regarding the level of support, both family members (such as a spouse, parents, etc.) and social support (medical policy support, friendly social environment, etc.) would increase medication adherence in patients with schizophrenia and also reduce patients’ stigma to a certain extent, especially for patients recovering from the illness. Positive treatment attitude and behavior, including seeing a doctor in time when the disease occurs, insisting on psychological intervention and actively learning related knowledge, etc. These are helpful to the compliance of taking medicine, and at the same time, better guarantee the exertion of drug effect. This is consistent with a previous reanalysis of 38 control studies by Pitsche-l Walz et al., which showed that psychological education can improve the medication compliance of schizophrenia patients, and its one-year recurrence rate can be reduced by 20%^11^.

Generally speaking, medication adherence is the basic guarantee for schizophrenic patients to obtain the curative effect, which can better help patients recover and reduce recurrence, and then reduce the economic burden on family and society. The results of this meta-analysis may help identify low adherence to medication in patients with schizophrenia and inform the development of prevention protocols. Specifically, the results of the above five risk factors suggest that we have substance abuse behavior, low income, and low life satisfaction, age <25 or age >50, females with low education level are more likely to have drug non-adherence behavior, and we should focus on monitoring the population with the above characteristics. At the same time, combining the results of the two main protective factors we found that interventions targeting the problem of medication adherence can be aimed at improving patients’ life satisfaction, level of support, developing positive attitudes and behaviors, and educating about medication adherence as entry points.

## Supplementary information

SUPPLEMENTAL MATERIAL
