# Supplementary material for: Influencing factors of medication adherence in schizophrenic patients: a meta-analysis
Source: Schizophrenia (Heidelb). 2023 May 15;9(1):31. doi: 10.1038/s41537-023-00356-x (PMC10185672; doi:10.1038/s41537-023-00356-x)
Supplement: Supplementary file 1 — SUPPLEMENTAL MATERIAL [file 41537_2023_356_MOESM1_ESM.pdf]

Supplemental Table 1. Quality assessment of included cross-sectional studies (Agency for Healthcare Research and Quality)

Study	Publication year	1) Define the source of information (survey, record, review)	2) List inclusion and exclusion criteria for exposed and unexposed subjects (cases and controls) or refer to previous publications	3) Indicate time period used for identifying patients	4) Indicate whether or not subjects were consecutive if not population-based	5) Indicate if evaluators of subjective components of study were masked to other aspects of the status of the participants	6) Describe any assessments undertaken for quality assurance purposes (e.g., test/retest of primary outcome measurements)	7) Explain any patient exclusions from analysis	8) Describe how confounding was assessed and/or controlled.	9) If applicable, explain how missing data were handled in the analysis	10) Summarize patient response rates and completeness of data collection	11) Clarify what follow-up, if any, was expected and the percentage of patients for which incomplete data or follow-up was obtained	Quality score
Desai, R.	2019	1	1	1	1	0	1	1	0	1	1	0	8
Wang, D.	2020	1	1	1	1	0	1	1	0	0	1	0	7
Stockbridge, E. L.	2021	1	1	1	1	0	1	1	1	0	1	0	8
Verdoux, H.	2021	1	1	1	1	0	1	0	0	0	1	0	6
Kirchner, S. K.	2022	1	1	1	1	0	1	1	0	0	1	0	7

Notes: 1 means the study meets this criterion, 0 means the study does not meet this criterion. Quality score no less than 5 will be included in this meta-analysis.

Supplemental Table 2. Quality assessment of included longitudinal studies (Newcastle Ottawa Scale)

Study	Selection				Comparability	Outcome			Quality score
	Representativeness of exposed cohort	Selection of non-exposed cohort	Ascertainment of exposure	Demonstration that outcome of interest was not present at start of study	Comparability of cohorts on the basis of the design or analysis	Assessment of outcome	Was follow-up long enough for outcomes to occur (mean follow- up)	Adequacy of follow up of cohorts	
Olfson, M. 2000	1	0	0	1	1	1	0	1	5
Acosta, F. J. 2009	1	0	1	1	1	1	1	1	7
Gearing, R. E. 2009	1	0	1	1	1	1	1	1	7
Hill, M. 2010	1	0	0	1	1	1	1	1	6
Lambert, M. 2010	1	0	1	1	1	1	1	1	7
Conti, V. 2012	1	0	0	1	1	1	1	1	6
Hui, C. L. M. 2016	1	0	1	1	1	1	0	1	6
Anderson, J. P. 2017	0	0	1	1	1	1	1	1	6
Conus, P. 2017	1	0	0	1	1	1	1	1	6
Daneault, J. G. 2019	1	0	1	1	1	1	1	1	7

Rezansoff, S. N. 2019	1	0	1	1	1	1	1	1	7
Tan, C. Z. 2019	1	0	1	1	1	1	1	1	7
Guitter, M. 2021	1	0	0	1	1	1	1	1	6
Rubio, J. M. 2021	1	0	0	1	1	1	1	1	6
Cho, S. J. 2022	1	0	0	1	1	1	0	1	5

Notes: 1 means the study meets this criterion, 0 means the study does not meet this criterion. Quality score no less than 5 will be included in this meta-analysis.

Supplemental Table 3. Heterogeneity test - country subgroups

**Country**	***P***	I^2 (%)
Canada	0.001	99.1
Australia	0.001	78.3
United States	0.001	83.7
Sent	0.001	76.0
China	0.001	88.0
Germany and Australia	0.267	18.8
Spain	0.183	43.6
Ireland	0.001	95.5
Finland	0.001	94.7
Singapore	0.017	75.5
South Korea	0.001	99.6
Italy	0.001	90.5
Australia	0.001	95.6
